# The effects of increased therapy time on cognition and mood in frail patients with a stroke who rehabilitate on rehabilitation units of nursing homes in the Netherlands: a protocol of a comparative study

**DOI:** 10.1186/1471-2318-14-68

**Published:** 2014-05-23

**Authors:** Marleen Huijben-Schoenmakers, Arno Rademaker, Peter van Rooden, Erik Scherder

**Affiliations:** 1 Department of Clinical Neuropsychology, Free University of Amsterdam, Van der Boechorststraat 1, Amsterdam 1081 BT, The Netherlands

**Keywords:** Stroke, Rehabilitation, Cognition, Mood, Activities of daily living, Clinical nursing rehabilitation stroke guideline

## Abstract

**Background:**

Recovery after stroke is dependent on how much time can be spent on rehabilitation. Recently, we found that therapy time for older stroke patients on a rehabilitation unit of a nursing home could be increased significantly from 8.6 to at least 13 hours a week. This increase was attained by the implementation of interventions, focused on strength, mobility and balance. Nurses carried out these exercises with the patients during their daily activities. The aim of the present study is to investigate if increased therapy time has a positive effect on cognition, mood (depression and anxiety), and ADL in stroke patients.

**Methods:**

A comparative single blind controlled study will be applied. Patients suffering from a stroke and staying on one of the rehabilitation units of the nursing homes are eligible for participation. Participants belong to the intervention group if they stay in two nursing homes where four interventions of the Clinical Nursing Rehabilitation Stroke Guideline were implemented. Participants who stay in two nursing homes where therapy is given according to the Dutch stroke Guideline, are included in the control group. Clinical neuropsychologists will assess patients’ cognitive functioning, level of depression (mood) and anxiety. Nurses will assess a Barthel Index score on a weekly basis (ADL). These variables are measured at baseline, after 8 weeks and at the moment when participants are discharged from the nursing home.

**Discussion:**

The present study evaluates the effect of increased therapy time on cognition, mood (level of depression and anxiety), and ADL in stroke patients. When positive effects will be found this study can guide policy makers and practitioners on how to implement more therapy time on rehabilitation wards of nursing homes.

**Trial registration:**

TNR Our study has been documented in the Dutch Trial Registration, TC = *3871*.

## Background

Recovery from stroke is highly dependent on intensive therapy executed by a multidisciplinary team [1-7]. Surprisingly, in a previous study we found that time spent on non-therapeutic activities accounted for 80% of the daytime, of which 28% was spent on sitting [8]. These undesirable results are supported by other studies examining the amount of time a patient actively participates in rehabilitation on a stroke unit and in hospitals [9-15]. In a recent study we showed that therapeutic activities at a rehabilitation unit of a nursing home can be increased with more than 50 minutes a day when nurses built in exercises during their daily routines [16]. In this way patients perform 50% more therapeutic activities during the day compared to what is prescribed by the Dutch Stroke Guidelines [17].

There is ample evidence that exercise has a beneficial influence on, among others, cognitive functions, in older persons without dementia [18].

Cognitive functions that respond positively to exercise are executive functions in particular [19]. Executive functions include higher-order cognitive functions such as planning, set-shifting, attention, impulse control, and working memory [20]. A prerequisite is that these older persons are sedentary at the beginning of the exercise program [21]. There is a limited number of studies showing improvement of cognitive functioning in stroke patients as a consequence of exercise. Some cognitive domains related to motor learning, e.g. information processing speed and attention improved by aerobic exercise post stroke [22,23]. It is interesting to know if other impaired domains of cognition in stroke patients can be influenced by exercise, e.g. executive functions and memory as particularly these functions are crucial for independent functioning [24].

The question arises whether an increase in exercise time of approximately 50 minutes a day is sufficient to have a beneficial influence on cognition, e.g. executive functions, and mood in frail stroke patients. It is known that a decline in cognition and mood both relate to more severe functional limitations and less functional recovery [25,26]. More specifically, the prevalence of cognitive disorders in stroke patients is high and varies from 22% [27] to 63% [28]. It has been observed that cognitive impairment after stroke influences recovery of functional outcome and activities of daily living negatively in older stroke survivors [29,30]. Concerning mood, the frequency of e.g. depression after stroke is also high (33%) [31]. Depressed patients have poorer recovery patterns and need more time to achieve a high level of independence [32]. Moreover, stroke patients who could not successfully being discharged from nursing homes were more depressed [33].

In sum, the primary goal of the present study is to investigate whether an increase in therapy time has a beneficial influence on the various dimensions of cognition of frail stroke patients at discharge of rehabilitation units of nursing homes. The secondary goal is to examine whether an increase in therapy time improves mood, resulting in an improvement in activities of daily living (ADL).

## Methods

### Study design

The design of the study is a single blind controlled trial with two patients groups coming from nursing homes with two different rehabilitation approaches. The study consists of an effect evaluation and will be carried out in four nursing homes in the Netherlands. In the two control nursing homes, rehabilitation is only performed by therapists and based on the Dutch Stroke Guidelines [17]. Consequently, therapy time will be limited. In two intervention nursing homes, exercises derived from the Clinical Nursing Rehabilitation Stroke Guideline [34], are an additional part of the rehabilitation program, resulting in an extra 50 minutes a day of exercises for the patients.

The Medical Ethical Committee of the Free University Amsterdam, The Netherlands, approved this study.

### Study population

Patients suffering from a stroke will be eligible for participation, even if they suffer from co-morbid diseases such as heart disease, recurrent stroke or pre-existing depression. Patients will be excluded if they will leave the rehabilitation unit within two weeks, when they are too ill to participate, if there is a language problem, like aphasia, or when a Mini-Mental State Examination (MMSE) [35] score is lower than 13. A score of 13 or higher is a prerequisite for a neuropsychological assessment. Patients will be recruited by the head of the nursing staff, working on the rehabilitation unit of the nursing homes. Oral and written information about the study is given to the patients and/or their legal representatives. Prior to their participation in the study oral and written informed consent will be acquired from all the participants, their family or legal representatives. Informed consent will be based on veracity, anonymity, privacy, and confidentiality.

### Randomisation

A convenience sample will be taken of patients who rehabilitate in the selected nursing homes in the period of April 2012 until April 2015 and are willing to participate.

### Single-blind

The nursing homes, patients, caregivers, and the multidisciplinary teams who are participating in the study are not blinded for the treatment. Trained clinical neuropsychologists, collecting the data, do not belong to the permanent staff of the nursing homes and are not informed about the study objectives. Research assistants involved in the data analyses are blinded for the patients treatment background.

### Sample size

The sample size has been calculated by using the statistically power analysis program G Power. The estimated effect-size comes from a study reporting the effect of physical activity on cognition in patients with stroke (*d’* = 0.30) [27]. Taking into account a type one error of 0.05 (α = 0.05), a power of 0.80 (β = 0.08), three repeated measurements and two groups, a sample size of 138 patients is required, i.e. 69 persons in each group. Taken a dropout of 10% into account, a sample size of 81 persons in each group is required.

### Assessment

#### Measurement of extra therapy time

The difference between the intervention and the control nursing homes is the input of nurses resulting in extra exercises. In this way patients can increase their time spent on therapeutic activities. This is measured by Behavioural Mapping method [10,14,15]. Behavioural Mapping is a time sampling technique that provides registration of systematic and accurate observations resulting in therapeutic activities, nontherapeutic activities, interaction with others, and locations where activities take place. Observation of patients take place at 10- minutes intervals on weekdays (8:30–17:10). At each time point the observer records patient activity, the person attending to the patient and the patient’s location. In accordance to other studies the assumption is made that between two consecutive observations the three observation domains remain unchanged [10-15].

### Outcome measures

#### Patient characteristics

The following background characteristics are measured in both patient groups: age, gender, level of education, marital status, living situation, smoking and drinking alcohol.

### Medical history data

Type of stroke, TIA, Parkinson, disorders of consciousness, brain tumor, epilepsy, alcohol or other abuse, depression and or psychiatric problems and medication. Medication use is coded according to the guideline of the Dutch Pharmacotherapeutic Compass [36].

### Effect evaluation

#### Primary outcome measures cognition

##### Assessment of cognition

Cognition will be assessed by the following neuropsychological tests:

### Global cognitive functioning

#### Mini-mental state examination

Global cognitive functioning will be assessed by the Mini-Mental State Examination (MMSE). MMSE is a brief screening test and has been developed to screen for cognitive impairment, which has a joint reliability of 0.95 in a study of patients with various neurological disorders [35].

### Executive functions

#### Behavioural assessment of dysexecutive syndrome

##### Rule Shift Cards

A subtest of the BADS [37] is the Rule Shift Cards (BADS-RS), which is meant to assess set-shifting. This subtest discriminates people with an executive dysfunction from healthy people. Participants have to respond to stimuli (red or black playing cards) according to two rules that are presented consecutively. The maximum score is 4.

##### *Key-search test*

Another subtest of the BADS is the Key-search test. This test assesses the executive sub functions planning and problem-solving. The goal is to choose a systematic, and efficient strategy to find a key lost in a field. The inter-rate reliability of the BADS varies from 0.88-1.00. Brain injured patient achieve significant lower scores on the test which suggest that construct validity of these tests is as good as that of established tests [37]. The maximum score is 4.

#### Category fluency

Set-shifting, one of the executive functions, will be assessed by Category Fluency [38,39]. This test requires shifting from one category of names to another category. This test uses the category ‘animals’, test re-test reliability 0.82, and professions, test re-test reliability 0.96.

### Verbal and non-verbal memory

#### ‘Eight words test’

The patient is asked to memorize 8 words which have to be reproduced by the participant in different settings at 5 consecutive times with different time delays [40]. The maximum score of the Eight words test is 40.

#### Rivermead behavioural memory test

Episodic memory will be tested by the Rivermead Behavioural Memory Test (RBMT) Face and Picture Recognition [41]. The Face Recognition subtest is meant to assess nonverbal episodic memory; the Picture Recognition subtest is meant to assess both verbal and nonverbal episodic memory. The RBMT has reliability coefficients ranging from 0.57 to 0.86, with the inter scorer reliability of the RBMT being 0.9 or higher. The maximum score of the Face Recognition subtest is 10. The maximum score of the Picture Recognition subtest is 20 [41].

### Executive functions combined with memory

#### Montreal cognitive assessment

Montreal Cognitive Assessment (MOCA) assesses executive functions, attention and concentration, memory, language, orientation, calculation, conceptual thinking, and visuoconstructional skills [42]. The MOCA detects mild cognitive impairment with 90%-96% sensitivity and 87% specifity [42].

### Secondary outcome measure

#### Mood

The level of depression and anxiety will be assessed by the following questionnaires:

#### Beck depression inventory (BDI)

The BDI is a 20-item questionnaire used to measure the level of depression, with a high reliability (Coefficient Alpha = .86) [43]. A low score indicates a low level of depression [43]. The maximum score of the BDI is 60.

#### Symptoms checklist 90 (SCL90)

This test is a short test to examine anxiety and depression [44]. The scale exists of 25 questions about anxiety and depression, with an internal consistency coefficient rating range of .79 for paranoid ideation to .90 for depression. A higher score indicates a higher level of depression and anxiety. The maximum score of the SCL90 is 125 [44].

### Activities of daily life

Activities of daily life are measured by the Barthel Index [45].

#### Barthel index

The Barthel Index consists of 10 items, all focused on ADL activities. The total score of the Barthel Index ranges between 0 and 20 points. The Barthel Index has a high level of reliability and validity in patients with stroke [45].

### Intervention

#### Additional clinical nursing rehabilitation stroke guideline

##### Control group

Patients in the control nursing homes, receive approximately four hours a week of traditional rehabilitation based on the Dutch Stroke Guideline [17].

##### Experimental group

During their stay, patients in the intervention group, get extra therapy based on the Clinical Nursing Rehabilitation Stroke Guideline: muscle strengthening, sitting balance and reach, getting up from a chair and walking [34]. Nurses perform all exercises during the daily activities of the patients, including weekends, resulting in an extra therapy time of approximately five hours a week compared to the control group. These exercises are adapted to the patient‘s individual goals, need and rehabilitation level by physiotherapists and occupational therapist in consultation with the responsible nurses. Exercises will be documented in an exercise map which is fixed to the bed or wheelchair of the patient. Nurses coordinate the weekly adjustment exercises by informing the multidisciplinary team about the patient’s progress.

### Procedure

Within the first week of stay at the stroke units, eligible patients are tested by clinical neuropsychologists. They assess patient’s cognition and mood. These variables are measured at baseline, after 8 weeks and at discharge from the nursing home where the patient rehabilitates. In practice the moment of discharge can vary depending on the patient’s condition and personal situation. Therefore when patients are discharged earlier than 8 weeks the moment of discharge is selected as post measurement.

Nursing staff collects patients background characteristics at entry of the study and assesses a Barthel Index score every week. A geriatric physician collects medical history data.

The control group receives exercise therapy provided by physiotherapists or occupational therapists, once a day during 30 minutes on weekdays, but not in weekends. The intervention group receives the same therapy as the control group. Additionally, patients in the intervention group are stimulated, helped and controlled by nurses to carry out exercises from their exercise map every day including weekends. This results in approximately 10 to 20 extra exercise moments a day for these patients.

### Data-analysis

Descriptive analysis on all the patient characteristics at baseline is performed. A t-test for independent groups will be used to check for comparability between the intervention and control group at baseline. A repeated-measures multivariate analysis of variance (MANOVA) will be applied to assess possible effect of increased therapeutic time over various test moments (baseline, 8 weeks and at the moment of discharge). The level of significance is 0.05. The SPSS-PC version no. 20 will be used for data-analyses (SPSS Inc., Chicago, IL, USA).

## Discussion

The present protocol paper describes the design of a single blind controlled trial. The aim of our study is to investigate whether additional therapy time focused on muscle strengthening, sitting balance and reach, getting up from a chair and walking has a positive effect on cognition, mood and activities of daily living (ADL) in stroke patients who rehabilitate on rehabilitation units of nursing homes. The study gives insight into how the care of elderly stroke patients in nursing homes might be improved so that patients have a better chance of independent living. When positive effects will be found this study can guide policy makers and practitioners on how to implement higher quality care on rehabilitation wards of nursing homes.

The strength of the study is that 1) interventions from the evidence based Clinical Nursing Rehabilitation Stroke Guideline are implemented in the rehabilitation care of older stroke patients. This implementation will significantly increase the time spent on therapeutic activities without extra therapists needed, 2) the role of nurses can shift towards a more therapeutic one, 3) we examine if an increase in therapeutic time has a beneficial effect on cognition, mood, and ADL. These factors are of great importance for rehabilitation success in stroke patients. Possible limitations of our study are 1) the high rate of drop-out due to illness or death of this frail patient group, 2) the convenience sample causing a possible selection bias, 3) the limited intervention time before patients are discharged from the rehabilitation units.

## Competing interests

The authors declare that they have no competing interests.

## Authors’ contributions

MH-S has drafted the manuscript and performs the study. AR critically revised the manuscript and analyses data with SPSS. PvR has described the neuropsychological tests. Finally ES has designed the study and critically revised the manuscript. All authors read and approved the final version of the manuscript. MH-S, AR and ES revised the final version of the manuscript.

## Pre-publication history

The pre-publication history for this paper can be accessed here:

http://www.biomedcentral.com/1471-2318/14/68/prepub
